# Temperature based Restricted Boltzmann Machines

**DOI:** 10.1038/srep19133

**Published:** 2016-01-13

**Authors:** Guoqi Li, Lei Deng, Yi Xu, Changyun Wen, Wei Wang, Jing Pei, Luping Shi

**Affiliations:** 1 Center for Brain Inspired Computing Research, Department of Precision Instrument, Tsinghua University, Beijing, China, 100084; 2 School of Computing Enginering, Nanyang Technological University, Singapore, 639798; 3 School of Electrical and Electronic Engineering, Nanyang Technological University, Singapore, 639798; 4 School of Automation Science and Electrical Engineering, Beihang University, Beijing, China, 100191

## Abstract

Restricted Boltzmann machines (RBMs), which apply graphical models to learning probability distribution over a set of inputs, have attracted much attention recently since being proposed as building blocks of multi-layer learning systems called deep belief networks (DBNs). Note that temperature is a key factor of the Boltzmann distribution that RBMs originate from. However, none of existing schemes have considered the impact of temperature in the graphical model of DBNs. In this work, we propose temperature based restricted Boltzmann machines (TRBMs) which reveals that temperature is an essential parameter controlling the selectivity of the firing neurons in the hidden layers. We theoretically prove that the effect of temperature can be adjusted by setting the parameter of the sharpness of the logistic function in the proposed TRBMs. The performance of RBMs can be improved by adjusting the temperature parameter of TRBMs. This work provides a comprehensive insights into the deep belief networks and deep learning architectures from a physical point of view.

A restricted Boltzmann machine (RBM) is a generative stochastic artificial neural network^1^,^2^,^3^,^4^,^5^,^6^ that applies graphical models to learning a probability distribution over a set of inputs^7^. The restricted Boltzmann machines (RBMs) were initially invented under the name Harmonium by Smolensky in 1986 8. After that, Hinton *et al.* proposed fast learning algorithms for training a RBM in mid-2000s^9^,^10^,^11^,^12^. Since then, RBMs have found wide applications in dimensionality reduction^9^, classification^13^,^14^,^15^,^16^,^17^,^18^, feature learning^19^,^20^,^21^,^22^,^23^,^24^,^25^, pattern recognition^26^,^27^,^28^,^29^, topic modelling^30^ and various other applications^31^,^32^,^33^,^34^,^35^,^36^,^37^,^38^,^39^. Generally, RBMs can be trained in either supervised or unsupervised ways, depending on the task. RBMs originate from the concept of Boltzmann distribution^40^, a well known concept in physical science where temperature is a key factor of the distribution. In fact, in statistical mechanics^41^,^42^,^43^ and mathematics, a Boltzmann distribution is a probability distribution of particles in a system over various possible states. Particles in this context refer to gaseous atoms or molecules, and the system of particles is assumed to have reached thermodynamic equilibrium^44^,^45^. The distribution is expressed in the form of 
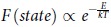
 where *E* is state energy which varies from state to state, and *kT* is the product of Boltzmann’s constant and thermodynamic temperature. However, none of existing schemes in RBMs consider the temperature parameter in the graphical models, which limits the understanding of RBM from a physical point of view.

In this work, we revise the RBM by introducing a parameter *T* called “temperature parameter”, and propose a model named “temperature based restricted Boltzmann machines” (TRBMs). Our motivation originates from the physical fact that the Boltzmann distribution depends on temperature while so far in RBM, the effect of temperature is not considered. The main idea is illustrated in Fig. 1. From a mathematical point of view, the newly introduced *T* is only a parameter that gives more flexibility (or more freedom) to the RBM. When *T* = 1, the model TRBM reduces to the existing RBM. So the present RBM is a special case of the TRBM. We further show that the temperature parameter *T* plays an essential role which controls the selectivity of the firing neurons in the hidden layers. In statistical mechanics, Maxwell-Boltzmann statistics^46^,^47^,^48^ describes the average distribution of non-interacting material particles over various energy states in thermal equilibrium, and is applicable when the temperature is high enough or the particle density is low enough to render quantum effects negligible. Note that the change in temperature affects the Maxwell-Boltzmann distribution significantly, and the particle distribution depends on the temperature (*T*) of the system. At a lower temperature, distributions moves to the left side with a higher kurtosis^49^. This implies that a lower temperature leads to a lower particle activity but higher entropy^50^,^51^,^52^. In this paper, we uncover that *T* affects the firing neurons activity distribution similar to that of a temperature parameter in Boltzmann distribution illustrated in Fig. 1, which gives some insights on the newly introduced *T* from physical point of view. From the figure, it is seen that a TRBM is a variant of the Boltzmann machine (BM) and RBM named after Boltzmann distribution. Note that the difference between BM and RBM lies in the various constraints on the connections between neurons, while the energy function in both BM and RBM follows the Boltzmann distribution. So far in both BM and RBM, the effect of temperature is not considered, especially when used for machine learning. In this work, we address such an issue by introducing a temperature parameter *T* into the probability distribution of the energy function following the Boltzmann distribution.

Our approaches and contributions are summarized as follows. Firstly, we prove that the effect of the temperature parameter can be transformed to the steepness of the logistic function^53^,^54^ which is a common “S” shape (sigmoid curve) when employing the contrastive divergence algorithm in the process of pre-training of a TRBM. Because the steepness of the sigmoid curve changes the accept probability when employing the Markov Chain Monte Carlo (MCMC) methods^55^,^56^ for sampling the Markov random process^57^,^58^. Secondly, it is proven that the error propagated from the output layer will be multiplied by 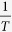, i.e., the inverse of the temperature parameter *T*, in every layer when doing a modified back propagation (BP) in the process of fine-tuning of a TRBM. We also show that the propagated error further affects the selectivity of the features extracted by the hidden layers. Thirdly, we show that the neural activity distribution impacts the performance of the TRBMs. It is found that the relatively lower temperature enhances the selectivity of the extracted features, which improves the performance of a TRBM. However, if the temperature is lower than certain value, the selectivity turns to deteriorate, as more and more neurons become inactive. Based on the results established in this paper, it is natural to imagine that temperature may affect the cognition performance of a real neural system.

## Results

### Temperature based Restricted Boltzmann Machines

As mentioned, a RBM is a generative stochastic artificial neural network that can learn a probability distribution over a given set of inputs. A RBM is a variant of the original Boltzmann machine, which requires all neurons to form a bipartite graph – neurons are divided into two groups, where one group contains “visible” neurons and the other group contains “hidden” ones. Neurons from different groups may have a symmetric connection, but there is no connection among neurons within the same group. This restriction allows for more efficient training algorithms which are available for the original class of Boltzmann machines, in particular the gradient-based contrastive divergence algorithm^59^,^60^.

It is well known that a RBM is an energy-based model in which the energy function^61^,^62^,^63^ is defined as


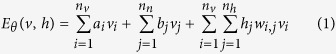


where *θ* consists of *W* = (*w*_*i*,*j*_) (size *n*_*v*_ × *n*_*h*_) which is associated with the connection between a hidden unit *h*_*j*_ and a visible unit *v*_*i*_, and bias weights *a*_*i*_ for visible units and *b*_*j*_ for hidden units. To incorporate the temperature effect into the RBM, a temperature parameter *T* is introduced to the following joint distribution of the vectors *v* and *h* of the “visible” and “hidden” vectors:


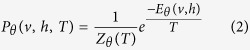


where *Z*_*θ*_(*T*), the sum of 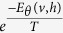 over all possible configurations, is a normalizing constant which ensures the probability distribution sums to 1, i.e.,


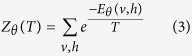


Denote ***v*** as an observed vector of *v*. Similar to RBM, TRBM is trained to maximize a distribution called likelihood function *P*_*θ*_(***v***), which is a marginal distribution function of *P*_*θ*_(*v*, *h*, *T*), i.e.,


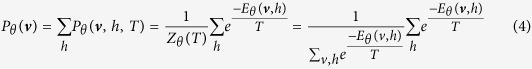


It is obtained that


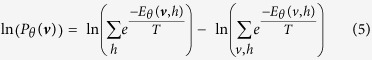


**Remark 1.** Similarly to RBM, there are two stages for training a typical TRBM for deep learning, i.e., a pre-training stage and a fine-tuning stage^10^,^11^,^12^. The most frequently used algorithms for these two stages are contrastive divergence and back propagation, respectively.

### Contrastive divergence for pre-training a TRBM

In the pre-training stage, the contrastive divergence algorithm performs MCMC/Gibbs sampling and is used inside a gradient descent procedure to compute weight update. Theorem 1 shows that we only need to modify the sharpness of a logistic function when temperature is considered, in order to employ the contrastive divergence algorithm.

***Theorem 1.*** When applying contrastive divergence for pre-training a TRBM, the temperature parameter controls the sharpness of the logistic sigmoid function.

***Proof.*** Note that for an observed ***v***, we have


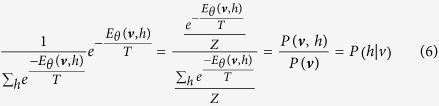


Then, the likelihood function can be written as


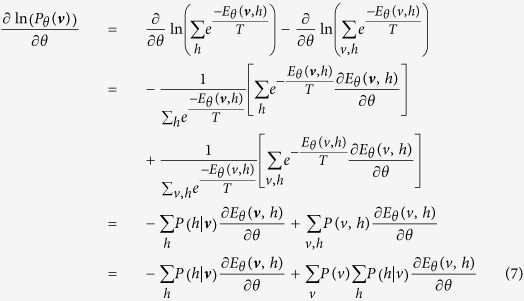


Denote that


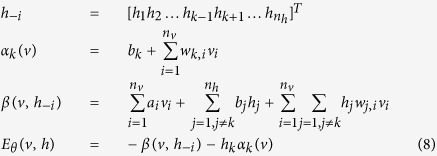


When *θ* = *W*_*ij*_, we have


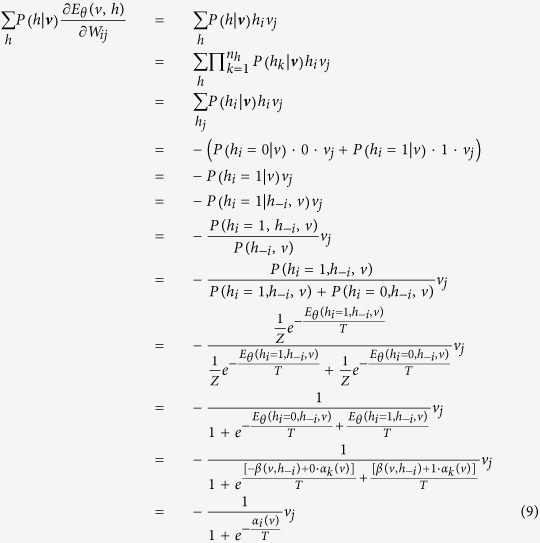


Then, it is obtained that


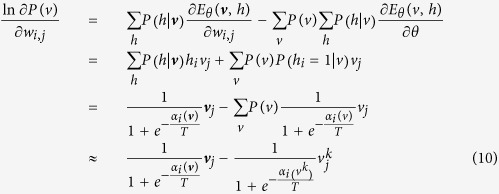


where 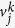 is the *k*–*th* Gibbs sampling of *v*_*j*_. Similarly, for the case that *θ* = *a*_*i*_ and *θ* = *b*_*i*_, we obtain that


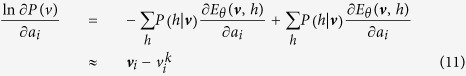


and


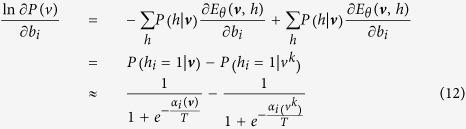


It can be seen that *T* actually controls the sharpness of the logistic function from equations (10) to (12), 21, 26, and the learning algorithm is given by:


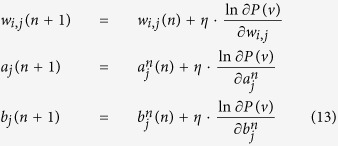


Thus, this theorem holds. □

Theorem 1 indicates that the effect of the temperature parameter can be effectively reflected on the sharpness of the logistic sigmoid function. This benefits the implementation of contrastive divergence in pre-training a TRBM as one only needs to adjust *T* as seen in equations (10)–(12), 21, 26.

### Back propagation for fine-tuning a TRBM

In employing the contrastive divergence algorithm for pre-training a TRBM, we have shown that the sharpness of the logistic sigmoid function reflects the temperature effection. In the fine-tuning stage, the back propagation will be applied. In this section, we further show how the temperature parameter affect the back propagation progress for fine-tuning a TRBM. It is shown that the error propagated from the output layer will be multiplied by 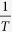 in every layer.

Let the logistic sigmoid function, which is also called the activation function, of the TRBM be


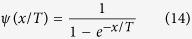


Note that 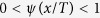 and the derivative of a sigmoid function is


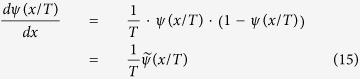


where 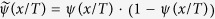 and we have 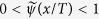.

***Theorem 2.*** When applying back propagation for fine-tuning a TRBM, the error signal propagated from the output layer of TRBM will be multiplied by 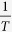 at every layer.

***Proof.*** From Fig. 2(a), when considering the gradient on the output layer, the cost function 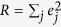, where *e*_*j*_ = *y*_*j*_ − *d*_*j*_, *d*_*j*_ is the output of the network and *y*_*j*_ is the given labels. We have


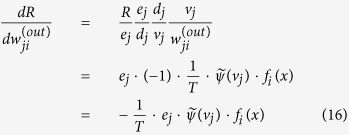


As shown in Fig. 2(b), suppose that the error signal on the layer *κ* which is transformed back from its next layer is fixed as 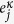, the cost function on layer *κ* can be regarded as 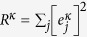. Then, the gradient of the cost function with respect to the weight 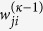 on layer *κ* − 1 by fixing the error signal 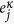 on layer *κ* is given by


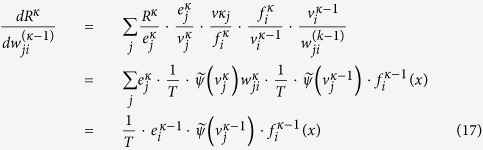


where


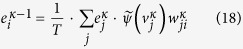


is the error signal for the neuron *i* on layer *κ* − 1, and it is the summarization of all the error signals transferred from layer *κ*. Comparing with the standard back propagation progress in RBMs, the error in doing back propagation in TRBM will be multiplied by 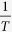, as summarized in Fig. 3. □

## Simulations

A 784-500-500-2000-10 network is built after layer-by-layer pre-training of TRBMs and overall fine-tuning under the back propagation algorithm. the training data is a MNIST set which contains 60000 handwritten digits. As there is no parameter *T* in a RBM and for the convenience of comparisons we introduce another parameter *T*_0_ set such that *T*/*T*_0_ = 1 corresponds the standard RBM. So the values of *T*/*T*_0_ reflect how we set the temperature in TRBM, i.e., a higher *T*/*T*_0_ implies a relative higher temperature compared with a RBM. We achieve 8 groups of weight parameters by training the network at different temperatures respectively, including *T*/*T*_0_ of 0.1, 0.2, 0.5 0.8, 1.0, 1.2, 1.5 and 2.0. For each testing, 10000 groups of sampling data of the firing neuron number in layer 2 are computed, in which the input for each sampling is randomly chosen from the 10000 digits in the MNIST testing set. Notably, the firing neuron is also a result of probabilistic sampling. For example, if the output of a neuron in layer 2 is 0.3, it has a chance of thirty percent to fire. At last, we illustrate the neuronal activity distribution via drawing the histogram of 10000 groups of the firing neuron number in layer 2. As the temperature gradually rises, the activity distribution curve moves to the right which indicates more firing neurons; while, the reverse result is observed when the temperature decreases.

Figure 4 shows the performance of the TRBMs with respect to different temperature values. We allow *T*/*T*_0_ changing from 0.1 to 2. Note that when *T*/*T*_0_ = 1, the model is reduced to the standard RBM. The temperature increases as *T*/*T*_0_ > 1, and decreases as *T*/*T*_0_ < 1. It is observed that the performance of the TRBM improves as the temperature decreases significantly when *T*/*T*_0_ is higher than 1. And it can be further improved when *T*/*T*_0_ becomes lower and achieves its best performance around *T*/*T*_0_ = 0.5. After that, the performance deteriorates as *T*/*T*0 decreases. The best error rate is around 1%.

To see how temperature affects the extracted features on layer 2, we plot these features in Fig. 5. Note that each neuron in layer 2 has a 784-dimensional weight vector as it connects 784 neurons in layer 1. We could reshape the 784-dimensional weight vector to a 28 × 28 matrix which is called a reconstruction map. As the reconstruction map can be reconstructed for each neuron in layer 2, all the maps we reconstructed are considered as extracted features on this layer. Figure 5 shows 15 × 15 randomly chosen reconstruction maps, where each block element corresponds to a reconstruction map of a neuron in layer 2. The reconstruction maps are obtained at four different temperatures: (a) *T*/*T*_0_ = 0.1; (b) *T*/*T*_0_ = 0.5; (c) *T*/*T*_0_ = 1.0; (d) *T*/*T*_0_ = 2.0. Four typical features are reconstructed, including blank, stroke, digit and snowflake. The blank feature indicates small weights and snowflake feature is resulted from large weights. As temperature arises, i.e., *T*/*T*_0_ increases, blank features become less while more snowflake features appear, which leads to intense responses of the post-neurons. Note that when *T*/*T*_0_ = 1.0 in Fig. 5(c), the TRBM is reduced to the standard RBM, where three features including stroke, digit and snowflake can be observed. But the TRBM captures the best stroke features at a relatively lower temperature *T*/*T*_0_ = 0.5 in Fig. 2(b), where the blank, the digit and the snowflake are almost invisible. However, as the temperature falls continuously, sparse activities^64^ of the neurons lead to better selectivity, but more inactive connections, i.e. blank features.

In addition, we show how the temperature affect the extracted features on the third layer in Fig. 6. Each neuron in layer 3 has a 500-dimensional weight vector connected to the neurons in layer 2, and each neuron in layer 2 has a weight reconstruction map. Consequently, we can reconstruct a weight map of each neuron in layer 3, by computing a weighted sum of all 500 reconstruction maps of the neurons in layer 2. Similarly, we obtain the extracted features on layer 3 at four different temperatures: (a) *T*/*T*_0_ = 0.1; (b) *T*/*T*_0_ = 0.5; (c) *T*/*T*_0_ = 1.0; (d) *T*/*T*_0_ = 2.0. Since the digit features are usually expected to appear at higher layers, here we focus on the number of digit features in the weight reconstruction map at different temperatures. As the temperature rises, the digit features significantly decrease. Considering the digit features in Figs 4 and 5 in the meantime, we conclude that a lower temperature accelerates the travelling speed of digit features from the input layer to the output layer. For example, when *T*/*T*_0_ = 0.1, the digit features begin to appear in the weight reconstruction map of the neurons in layer 2; while, this phenomenon is still not obvious even in the weight reconstruction map of the neurons in layer 2 when *T*/*T*_0_ = 2.

We also estimate the probability distribution of the number of firing neurons (or called “neuron activity distribution”) under different temperatures. Firstly, it is worthy to make clarifications on parameter *T* as the operation of TRBM is not the same as a physical heat exchange process. Since *T* can be considered as a mathematical parameter, it can be fixed as a constant when using the proposed modified back propagation (BP) algorithm to investigate its effects. In this case, the network runs by repeatedly choosing a unit and setting its state based on the training algorithm. According to a Boltzmann distribution, after running for sufficiently long time with a fixed *T*, the probability of a state of the network will depend only upon the final energy level of that state, and not on the initial state from which the process is started. This relationship indicates that the state distribution has converged to an equilibrium state. For example, as shown in Fig. 7 in the paper, if we train the network at a lower temperature *T*_1_, the probability distribution of the number of firing neurons (neuron activity distribution) will converge to an equilibrium state, i.e. the left-most curve; while if we increase the temperature to *T*_3_ when training, the state will converge to a new equilibrium state, i.e. the right-most curve. This implies that, the network state will reach a particular equilibrium distribution for a particular given parameter *T*. In other words, there exists a one-one mapping between *T* and the neuron activity distribution.

**Figure 1:**
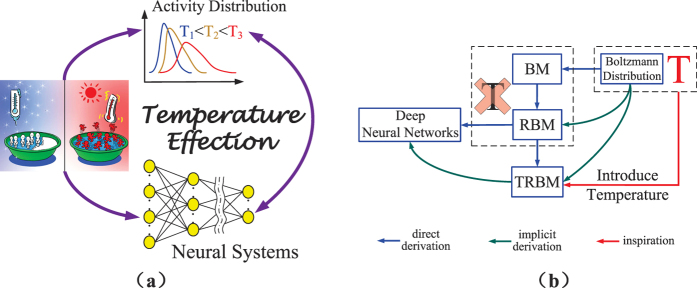
The main idea of this work. (**a**) The relationships among the temperature in a real-life physical systems, particle activity distribution and the artificial neural systems. (**b**) Illustration of a TRBM as a variant of Boltzmann machine (BM) and restricted Boltzmann machine (RBM).

**Figure 2:**
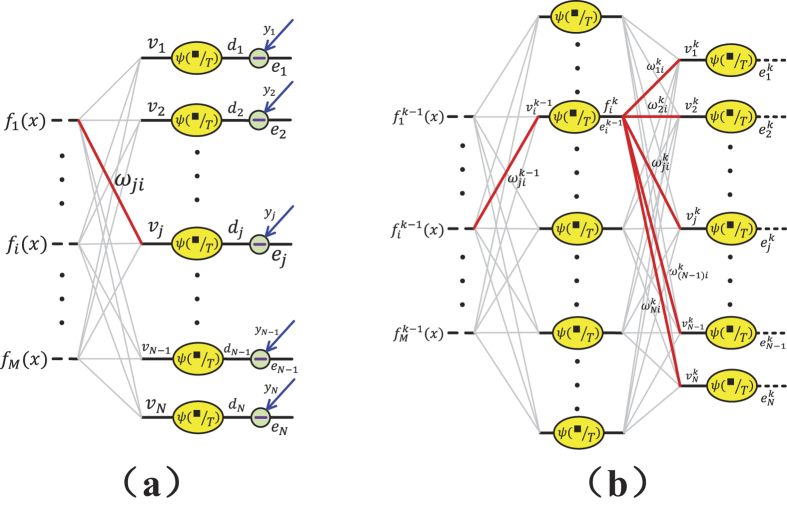
Illustration of the back propagation on TRBMs.

**Figure 3:**
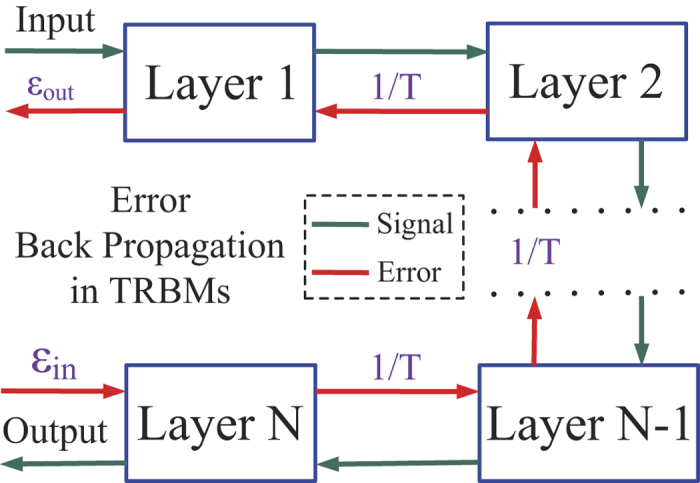
Illustrate of how temperature affects the back propagation progress. The error propagated from the output layer will be multiplied by 1/*T* in every layer. For a relative higher temperature since *T* > 1, the amplitude of the gradient will be reduced by 1/*T* times. For a relative higher temperature *T* < 1, the amplitude of the gradient will be strengthened by 1/*T* times.

**Figure 4:**
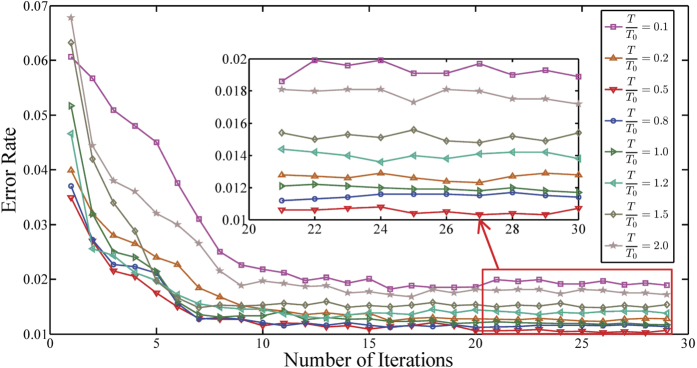
The performance of the TRBMs with respect to different temperatures. We choose 8 groups of weight parameters by training the network at different temperatures respectively, including *T*/*T*_0_ of 0.1, 0.2, 0.5 0.8, 1.0, 1.2, 1.5 and 2.0.

**Figure 5:**
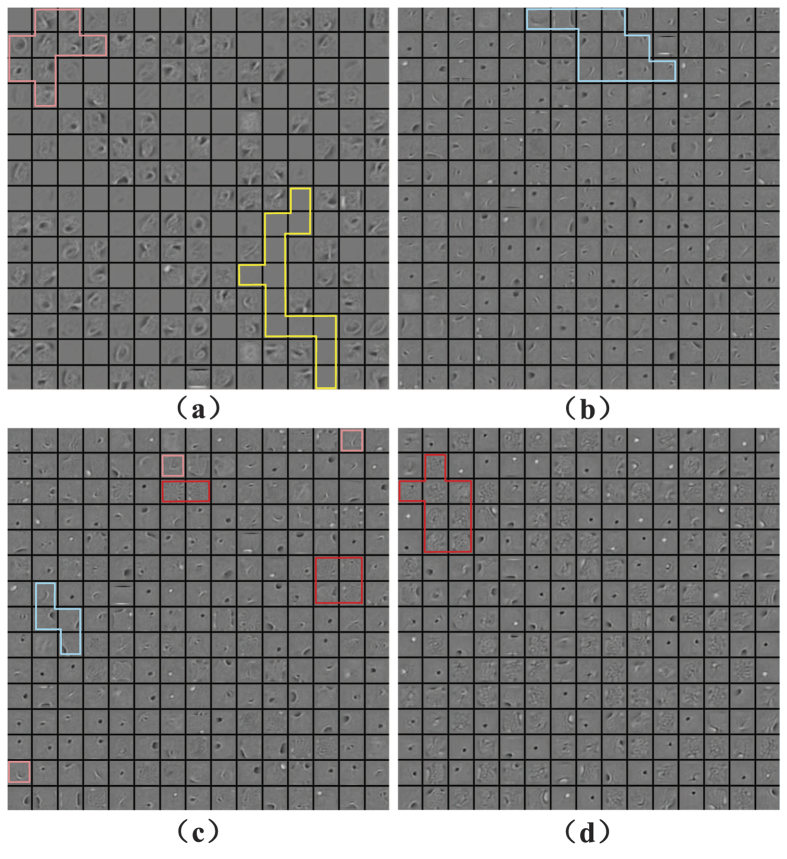
Temperature effection on the extracted features on layer 1 for (**a**) *T*/*T*_0_ = 0.1; (**b**) *T*/*T*_0_ = 0.5; (**c**) *T*/*T*_0_ = 1.0; (**d**) *T*/*T*_0_ = 2.0.

**Figure 6:**
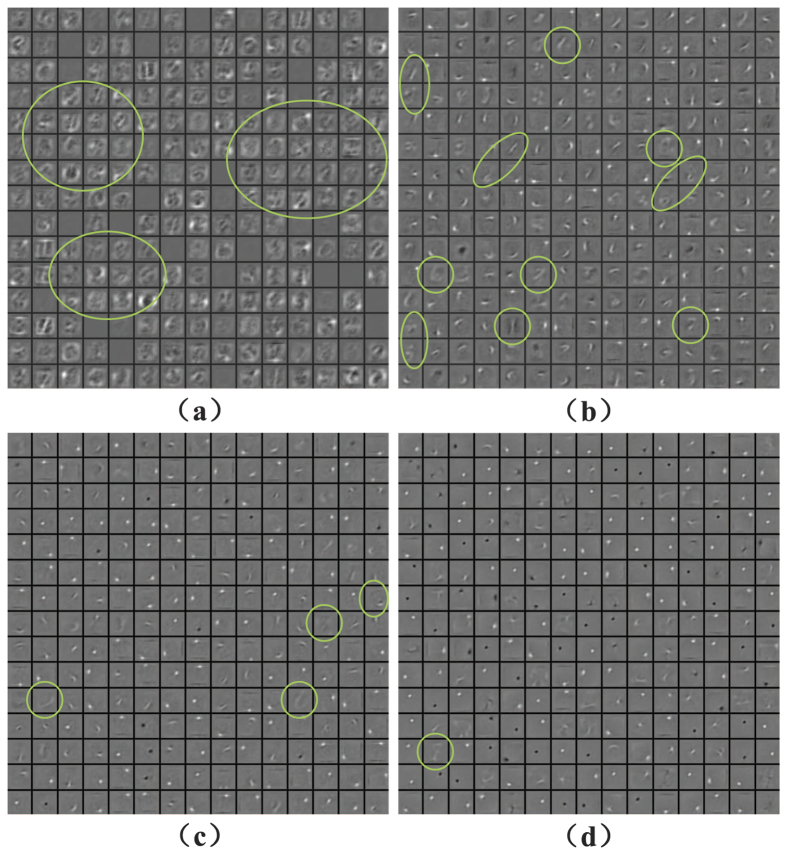
Temperature effection on the extracted features on layer 3 for (**a**) *T*/*T*_0_ = 0.1; (**b**) *T*/*T*_0_ = 0.5; (**c**) *T*/*T*_0_ = 1.0; (**d**) *T*/*T*_0_ = 2.0.

**Figure 7:**
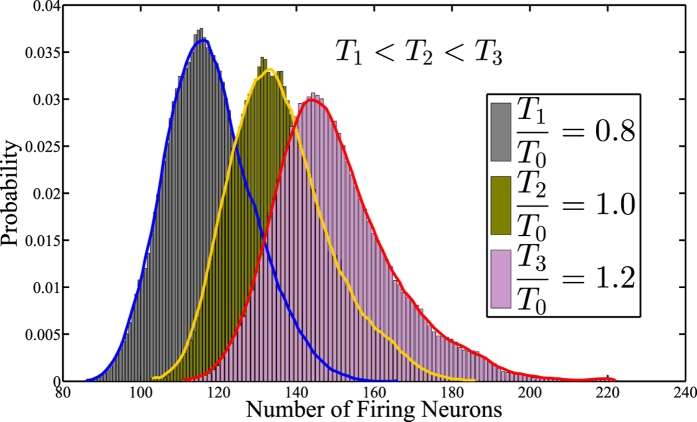
Estimated probability distribution of the number of firing neurons under different temperatures. The results are obtained by repeating the experiments with a different fixed *T* for each training process.

How *T* affects the firing neuron activity distribution can be tested by repeating experiments with a different fixed *T* for each training process with the results given in Fig. 7. It is observed that parameter *T* affects the firing neurons activity distribution similar to that of a temperature parameter in Boltzmann distribution illustrated in Fig. 1. Particularly, with lower temperature, the neuron activity decreases but with higher entropy and selectivity; while higher temperature leads to intense neuron activity. So the previous mentioned “equilibrium distribution” can be reasonably considered as a similar real-life thermal equilibrium. This is also why we could treat the parameter *T* as a temperature, and name our newly proposed RBM as Temperature based Restricted Boltzmann Machines (TRBM).

The kurtosis^65^ can be applied to characterize the selectivity of the TRBM, and it is the degree of peakedness of a distribution, defined as a normalized form of the fourth central moment of a distribution. Generally speaking, a higher degree of peakedness corresponds to a better selectivity. For a random variable *X* with mean and variance being *μ* and *σ*^2^, respectively, the kurtosis is defined by


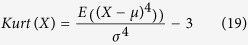


The “minus 3” at the end of this formula is often explained as a correction to make the kurtosis of the normal distribution equal to zero. It is observed that a higher temperature also leads to a more flat distribution, which has a lower kurtosis, i.e. poor selectivity. In contrast, better selectivity of low power often results in smaller error rate for pattern recognition. However, if the temperature is too low, the neuronal activity will be so sparse that the recognition results will become worse. In conclusion, for a relatively lower temperature, the distributions moves to the left side with a higher kurtosis, which implies that a lower temperature leads to a lower particle activity but a higher entropy. This is consistent with the observations on the neuron activity distribution in real-life physical systems in Fig. 1.

## Conclusions and Discussions

In this work, we propose a temperature based restricted Boltzmann machines which reveals that temperature is an essential parameter for controlling the selectivity of the firing neurons in the hidden layers. The two theorems we have established reveal the simplicity and applicability when implementing the proposed TRBM, because the effect of temperature can be efficiently adjusted by setting the sharpness of the logistic function, and the error propagated from the output layer only needs to be multiplied by 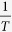 in every layer during the back propagation process. Clearly, our work brings more benefits to RBMs by bringing temperature into the model, such as more flexible choices, more completed and accurate modelling and results.

On the other hand, the work in this paper allows us to understand how temperature affects the performance of the TRBMs. It also stimulates our curiosity and opens our mind to think deeply about whether temperature affects the cognitive performance of real-life neural systems. An interesting study on cognitive performance of human beings in different seasons was conducted^66^, where tested subjects are required to respond to a visual stimulus with a key press as quickly as possible. Researchers found that the reaction of the subjects is faster in winter than in Summer, as they are more focused in cold weather. This interesting phenomenon consists with our observations in this paper, namely, relatively lower temperature improves the performance of a neural systems. Because the neural activity becomes thinner (higher kurtosis) in a relatively lower temperature environment, and thinner neural activity distribution makes the system have more feature selectivity ability. However, we know that more and more neurons will be inactive as the temperature decreases continually. Therefore, the performance improvement only exists in a narrow interval. The biological evolution has adjusted the neural systems to work well in a proper temperature range, which may have small fluctuations with respect to temperature. This provides a more comprehensive understanding on the artificial neural systems from a physical point of view, and may be essential for investigating the biological and artificial intelligence.

## Additional Information

**How to cite this article**: Li, G. *et al.* Temperature based Restricted Boltzmann Machines. *Sci. Rep.* **6**, 19133; doi: 10.1038/srep19133 (2016).
